# Pathologic Complete Response in Urothelial Carcinoma Patients Receiving Neoadjuvant Immune Checkpoint Inhibitors: A Meta-Analysis

**DOI:** 10.3390/jcm11041038

**Published:** 2022-02-17

**Authors:** Alessandro Rizzo, Veronica Mollica, Matteo Santoni, Gennaro Palmiotti, Francesco Massari

**Affiliations:** 1 Struttura Semplice Dipartimentale di Oncologia Medica per la Presa in Carico Globale del Paziente Oncologico “Don Tonino Bello”, I.R.C.C.S. Istituto Tumori “Giovanni Paolo II”, Viale Orazio Flacco 65, 70124 Bari, Italy; gennaropalmiotti@hotmail.it; 2 Medical Oncology, IRCCS Azienda Ospedaliero-Universitaria di Bologna, Via Albertoni—15, 40138 Bologna, Italy; veronica.mollica7@gmail.com (V.M.); fmassari79@gmail.com (F.M.); 3 Medical Oncology Unit, Macerata General Hospital, 62100 Macerata, Italy; mattymo@alice.it

**Keywords:** immunotherapy, immune checkpoint inhibitors, pembrolizumab, atezolizumab, urothelial carcinoma, neoadjuvant

## Abstract

Background. Immune checkpoint inhibitors (ICIs) have been evaluated as neoadjuvant treatment in urothelial carcinoma (UC) patients, with these agents reporting encouraging pathologic complete response (pCR) rates. Herein, we performed a systematic review and meta-analysis aimed at evaluating the incidence of pCR in UC patients treated with neoadjuvant ICI. Moreover, we investigated the impact of PD-L1 expression in this patient population, exploring the possible role of PD-L1 status as predictive biomarker. Materials and Methods. We retrieved all the relevant trials through PubMed/Medline, Cochrane Library and EMBASE; moreover, proceedings of the main international oncological meetings were also searched for relevant abstracts. Eligible trials assessed pre-operative ICI in UC patients. Results. Our meta-analysis has highlighted a pooled pCR rate of 36.6% in the overall population; interestingly, pCR was higher in PD-L1 positive compared with PD-L1 negative UCs (49.5% versus 35.1%, respectively). Conclusions. Positive signals emanating from neoadjuvant immunotherapy should encourage the scientific community to persist in the long road toward finding more effective treatments for UC patients.

## 1. Introduction

Urothelial carcinoma (UC) is the fourth most commonly diagnosed malignancy worldwide, representing an important cause of cancer-related death [1]; around 25–30% of patients present with muscle-invasive bladder cancer (MIBC) or metastatic UC at the time of diagnosis, while about 70–75% of cases are affected by non-muscle-invasive disease [2]. Since UC has been traditionally considered as an immunogenic tumor, immunotherapy has been tested in metastatic disease as well as in the adjuvant and neoadjuvant setting [3,4]. The advent of immune checkpoint inhibitors (ICIs) has represented a breakthrough in the first- and later-line setting of metastatic UC patients [5,6]. In fact, several clinical trials evaluating the role of immunotherapy in metastatic UC have reported practice-changing results, as also witnessed by the approval of five ICIs in the last few years, including pembrolizumab, nivolumab, atezolizumab, durvalumab, and avelumab, with the JAVELIN Bladder 100 phase III trial recently demonstrating that avelumab maintenance provides superior overall survival versus placebo in patients without disease progression with first-line chemotherapy [7,8].

In patients with localized UC, neoadjuvant cisplatin-based chemotherapy is frequently administered in everyday clinical practice, given the improvement in terms of overall survival reported in several studies and meta-analyses [9,10]. Of note, the use of cisplatin-based chemotherapy at the earliest point in the natural history of UC has the potential to maximize patient outcomes; however, most patients with localized disease and a plan to undergo radical surgery are not fit enough to receive neoadjuvant cisplatin due to underlying comorbidities and, as such, choosing the optimal management for this patient population remains challenging [11,12]. In fact, the perceived and real cisplatin-related adverse events are a major obstacle, and the UC medical community has focused its efforts towards the identification and development of more well-tolerated neoadjuvant treatments [13]. Among these, PD-1, PD-L1, and CTLA-4 inhibitors have been assessed as neoadjuvant treatment in UC patients, as monotherapy or in combination with other anticancer drugs, with these agents reporting encouraging pathologic complete response (pCR) rates in early phase clinical trials [14]. In addition, we have recently seen growing attention towards combination strategies, as witnessed by the presentation and publication of studies testing chemoimmunotherapy in cisplatin-eligible patients [15,16]. Thus, despite the use of neoadjuvant immunotherapy remaining investigational, the number of indications and UC patients receiving neoadjuvant ICIs is supposed to increase in the near future [17,18].

Herein, we performed a systematic review and meta-analysis aimed at evaluating the pooled incidence of pCR rate in UC patients treated with neoadjuvant ICIs. Moreover, we investigated the impact of PD-L1 expression in this patient population, exploring the putative role of PD-L1 status as predictive biomarker.

## 2. Evidence Acquisition

### 2.1. Search Strategies

All phase I, II and III clinical trials published from 10 June 2000 to 15 November 2021, were retrieved. Keywords used for searching on PubMed/Medline, Cochrane Library and EMBASE were: “immunotherapy” OR “nivolumab” OR “ipilimumab” OR “atezolizumab” OR “pembrolizumab” OR “durvalumab” OR “avelumab” OR “immune checkpoint inhibitors” AND “neoadjuvant treatment” OR “neoadjuvant therpay” AND “urothelial carcinoma” OR “bladder cancer” AND “bladder carcinoma”. Only articles published in peer-reviewed journals, with available full text, and written in English language were considered. Furthermore, proceedings of the main international oncological meetings (American Society of Clinical Oncology, American Association for Cancer Research, European Society of Medical Oncology, European Council of Clinical Oncology), were also searched from 2000 onward for relevant abstracts.

The search and review of the articles were evaluated by three authors independently.

### 2.2. Aims of the Systematic Review and Meta-Analysis

The aims of the systematic review and meta-analysis were:

- To evaluate the incidence rate of pCR in UC patients treated with neoadjuvant ICIs;
- To evaluate the incidence rate of pCR in PD-L1 positive and PD-L1 negative UC patients receiving neoadjuvant ICIs.

### 2.3. Selection Criteria

Studies selected from first analysis were then restricted to: (1) prospective phase I, II or III trials in UC; (2) participants treated with ICIs; (3) studies with available data about pCR; (4) studies with available data regarding pCR in PD-L1 positive and PD-L1 negative patients.

### 2.4. Data Extraction and Quality Assessment

The following data were extracted for each publication: (1) study information (author, phase, carry out country, inclusion criteria); (2) type and dose of ICI; (3) number of patients. Three separate authors conducted the search and identification independently.

We assessed the methodological quality of the included trials using Cochrane Collaboration tool. Studies evaluated were defined as having a “low risk”, “high risk”, or “unclear risk” of bias across the seven specified domains. The current analysis was conducted according to Preferred Reporting Items for Systematic Review and Meta-Analyses (PRISMA) guidelines (Supplementary Table S1) [19].

### 2.5. Assessment of Risk of Bias in Included Studies

Risk of bias in the selected studies was evaluated by three independent authors through the tool of The Cochrane Collaboration for assessing risk of bias and therefore including selection, performance, detection, attrition and reporting bias [20]. The lists of outcomes reported in the published paper were compared to those from study protocols or trials registries.

**Figure 1:**
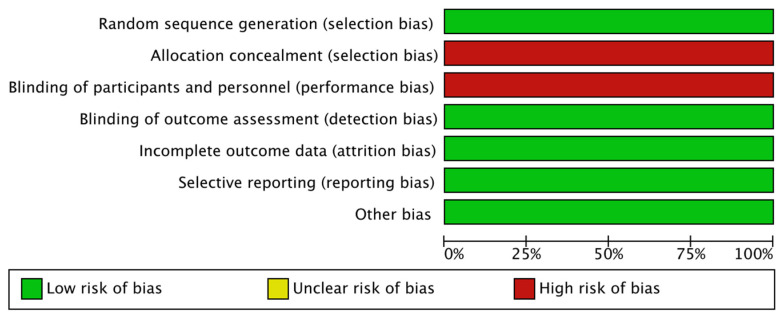
Risk of bias graph; authors’ judgements about each risk of bias item are presented as percentages across all included studies.

The results were summarized in a risk of bias graph (Figure 1).

### 2.6. Statistical Design

All statistical analyses were performed using R studio.

For the calculation of incidence rate, the number of patients (overall population, PD-L1 positive, and PD-L1 negative) with pCR receiving ICIs and the total number of patients being treated with ICIs were determined from each study. The proportions of patients and 95% Confidence Intervals (CIs) were derived.

## 3. Evidence Synthesis

### 3.1. Studies Selected

Table 1 reports a summary of the included studies [21,22,23,24,25].

**Figure 2:**
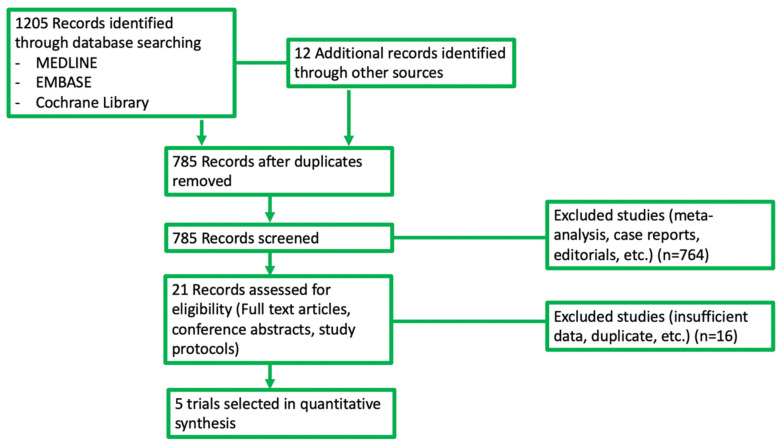
Diagram of all the trials included and excluded in the present meta-analysis.

In our search, we found 1217 potentially relevant reports, which were subsequently restricted to 5 [21,22,23,24,25]. We excluded 1212 records as non-pertinent reports (pre-clinical studies, retrospective studies, meta-analysis and systematic reviews, review articles, editorials, case reports, ongoing trials/trials in progress, non-randomized studies, no placebo-controlled arm trials), as shown in Figure 2.

### 3.2. Incidence Rate of Pathological Complete Response

In the overall population of UC treated with ICIs, the pooled incidence rate of pCR was 36.6% (95% CI, 30.3–41.4) (Table 2).

The pCR in PD-L1 positive and PD-L1 negative UC patients were 49.5% (95% CI, 38.8–60.6) and 35.1% (95% CI, 25.2–44.7), respectively (Table 2).

## 4. Discussion

To the best of the authors’ knowledge, the current study represents the first meta-analysis to provide a systematic evaluation of the pooled incidence of the pCR rate in UC patients receiving ICIs. Our findings further support the exploration of ICI-based strategies in this setting, where the results of ongoing phase III clinical studies on neoadjuvant immunotherapy are awaited. At the same time, our results suggest that the identification of specific histological and molecular predictors of response to neoadjuvant ICIs represents an important challenge in UC [26]. In fact, only part of UC patients seems to benefit from preoperative immunotherapy, highlighting the need for a deeper understanding of predictors of response. Selecting the most appropriate treatment remains complex, and positive signals emanating from neoadjuvant immunotherapy should encourage the scientific community to persist in the long road toward finding more effective treatments for UC patients.

Treatment paradigms of UC have seen important changes within a few years, and this rapidly changing landscape has prompted clinicians to consider the expansion of the role of ICIs to the earlier stages of the disease [27,28,29,30]. In fact, there is an urgent need to identify novel and active systemic treatments as part of pre-operative strategies, in order to attempt disease down-staging and to expand the proportion of UC patients that could derive benefit from surgery. In our analysis, we included five recent studies evaluating ICIs as neoadjuvant treatment in UC patients, including PURE-01, ABACUS, and NABUCCO [21,22,23,24,25]. In the PURE-01 trial, 50 patients were treated with pembrolizumab (200 mg, every three weeks) for three cycles before radical surgery [21]; the primary outcome measure was pCR rate, which was highlighted in 41% of patients in the intention-to-treat population [21]. Of note, PD-L1 positivity defined as Combined Positive Score (CPS) ≥ 10% was reported in 25 patients, which achieved pCR in 54% of cases, while this rate was considerably lower in PD-L1 negative subjects (13%) [21]. According to the results of this phase II trial, neoadjuvant pembrolizumab was well tolerated, with only 6% of UC patients reporting grade 3–4 treatment-related adverse events [21].

The ABACUS single-arm phase II trial evaluated the PD-L1 inhibitor atezolizumab (1200 mg, every three weeks) for two cycles in T2N0M0 (73%), T3N0M0 (19%), and T4N0M0 (8%) patients [22]; pCR rate was the primary endpoint of this study, occurring in 31% of subjects. Atezolizumab monotherapy was well tolerated, with 12% of patients experiencing any grade 3–4 toxicities [22]. Lastly, in the NABUCCO trial, the dual checkpoint blockade with the PD-1 inhibitor nivolumab plus the anti-CTLA-4 agent ipilimumab was investigated in 24 patients with stage III disease [23]; pCR was reported in 46% of cases. Compared to previously cited studies, grade 3–4 treatment-related adverse events were more common, since 55% of patients experienced these toxicities [23].

Our meta-analysis presents some noticeable limitations. In fact, the analyses were performed by extracting data from clinical studies, and individual patients’ data were not available; thus, confounding factors have not been included. Second, included trials were heterogeneous in terms of treatment arm as well as patient population and sample size, as reported in Table 1 [21,22,23,24,25]; in fact, despite the five immunotherapies (pembrolizumab, atezolizumab and nivolumab-ipilimumab) sharing several pharmacokinetic and pharmacodynamic features, these ICIs do not present superimposable effects [21,22,23,24,25]. Third, although the five studies present some common features, PD-L1 testing, and expression varied across different trials. For example, PD-L1 positivity was defined by PD-L1 CPS ≥ 10% in PURE-01 and NABUCCO and by Immune Cells (IC) ≥ 5% in ABACUS [21,22,23], and this element could have introduced some bias. However, our study has the merit of systematically assessing the role of neoadjuvant ICIs in this setting, suggesting a high pCR rate compared with historical data from previous clinical trials and meta-analyses, suggesting a pCR of approximately 25% in UC patients treated with neoadjuvant cisplatin-based chemotherapy, as also suggested by the recently presented VESPER V05 Phase III trial. In this setting, several questions remain unanswered, including the proper identification of biomarkers of response since a non-negligible proportion of UC patients does not benefit from neoadjuvant immunotherapy.

## 5. Conclusions

In the current study, we reported a promising pCR rate of 36.6% in all-comers, with this proportion rising to 49.5% in PD-L1 positive patients. The results of ongoing phase III trials evaluating neoadjuvant immunotherapy as monotherapy or in combination with other anticancer agents are awaited in order to confirm the encouraging results reported in phase I and II studies. Our findings support the exploration of ICI-based strategies as neoadjuvant treatments in UC, and the results of ongoing phase III clinical studies are highly awaited. The identification of histological and molecular predictors of response to neoadjuvant ICIs represents an important challenge, since only part of UC patients seems to benefit from preoperative immunotherapy.

## Figures and Tables

**Table 1:** Summary of trials included in the current study. Abbreviations: CPS: Combined Positive Score; IC: Immune Cells.

Trial	Phase	Stage	Neoadjuvant Treatment	Primary Endpoint	Number of Patients	PD-L1 Positive Definition	Safety
PURE-01	II	54% cT3 42% cT2 4% cT2-3N1	Pembrolizumab (three cycles)	Pathologic complete response rate	50	PD-L1 CPS ≥ 10%	The most frequent all-grade AE was thyroid dysfunction (n = 9; 18%), and there were three patients (6%) with grade 3 AEs that caused pembrolizumab discontinuation for one patient
ABACUS	II	73% cT2 19% cT3 8% cT4 0% N+	Atezolizumab (two cycles)	Pathologic complete response rate	95	PD-L1 IC ≥ 5%	Grade 3 or 4 Common Terminology Criteria (CTC) for adverse events (AEs) occurred in 10 of 95 (11%) patients
NABUCCO (cohort 1)	I	58% cT3-4a N0 42% cT2-4a N1-3	Nivolumab plus ipilimumab (two cycles)	Number of patients that have surgical resection < 12 weeks after study start	24	PD-L1 CPS ≥ 10%	Grade 3–4 immune-related adverse events occurred in 55% of patients and in 41% of patients when excluding clinically insignificant laboratory abnormalities.
NCT02812420	I	11% cT4 43% cT3 43% cT2 4% cT1	Durvalumab plus tremelimumab (two cycles)	Incidence of adverse events determined by extreme toxicity	28	PD-L1 IC ≥ 5%	6 of 28 patients (21%) presented grade ≥3 immune-related adverse events, consisting of asymptomatic laboratory abnormalities (n = 4), hepatitis and colitis (n = 2)
BLASST-2	I	100% cT2-T4aN0	Durvalumab	Number of Participants Receiving at least One Dose of Study Therapy Followed by Surgery without Dose-Limiting Toxicity (DLT) up to Twelve Weeks Post-Radical Cystectomy	10	PD-L1 IC ≥ 5%	One Grade 3 treatment-related adverse event (trAE) was reported (anemia), with no Grade 4 or higher trAE

**Table 2:** Pooled incidence of pathological complete response rate resulting from neoadjuvant immune checkpoint inhibitors treatment.

	Pathological Complete Response Rate % (95% CI)
Overall Population	36.6% (30.3; 41.4)
PD-L1 Positive Patients	49.5% (38.8; 60.6)
PD-L1 Negative Patients	35.1% (25.2; 44.7)

Abbreviations: CI: Confidence Interval.

## Data Availability

Not applicable.

## Supplementary Materials

The following supporting information can be downloaded at: https://www.mdpi.com/article/10.3390/jcm11041038/s1, Table S1: PRISMA Checklist.
